# Supplementary material for: Bone Turnover Markers, n-Terminal Propeptide of Type I Procollagen and Tartrate-Resistant Acid Phosphatase Type 5b, for Predicting Castration Resistance in Prostate Cancer
Source: Biomedicines. 2024 Jan 26;12(2):292. doi: 10.3390/biomedicines12020292 (PMC10887302; doi:10.3390/biomedicines12020292)
Supplement: Supplementary file 1 [file biomedicines-12-00292-s001.zip › Figure S1.pdf]

a

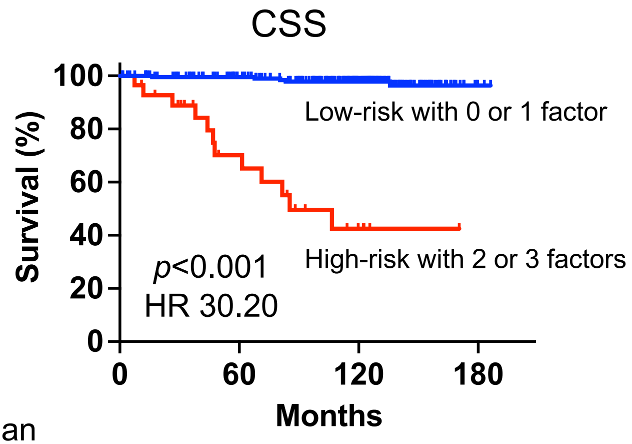

BGM classification	Median OS	Numbers at risk			
Low	NR	227	188	107	2
High	85.3	28	15	5	0

b

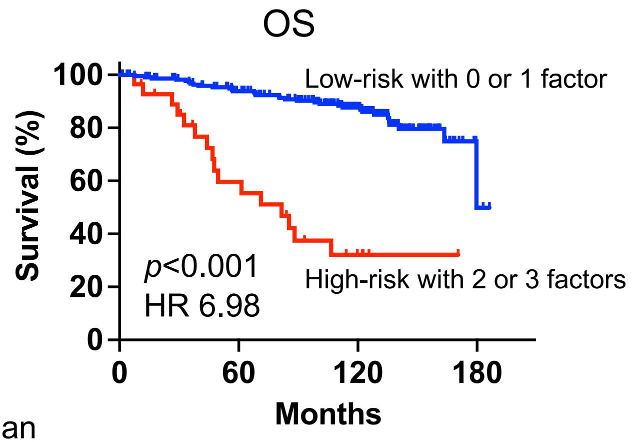

BGM classification	Median OS	Numbers at risk			
Low	179.7	227	188	107	2
High	81.6	28	15	5	0
